# Salivary alpha-amylase over cortisol ratio as a longitudinal indicator of work stress and psychosomatic strain in teachers

**DOI:** 10.3389/fendo.2025.1492379

**Published:** 2025-05-21

**Authors:** Ida Schneider, Alexander Wettstein, Gabriel Jenni, Fabienne Kühne, Martin grosse Holtforth, Sebastian Wachs, Roberto La Marca

**Affiliations:** ^1^ Department of Research and Development, University of Teacher Education Bern, Bern, Switzerland; ^2^ Clinical Psychology and Psychotherapy, Department of Psychology, University of Bern, Bern, Switzerland; ^3^ Psychosomatic Medicine, Department of Neurology, Inselspital, Bern University Hospital, University of Bern, Bern, Switzerland; ^4^ Digitalisation in Pedagogical Fields of Action, Institute of Education, University of Münster, Münster, Germany; ^5^ Clinica Holistica Engiadina, Susch, Switzerland; ^6^ Clinical Psychology and Psychotherapy, Department of Psychology, University of Zurich, Zurich, Switzerland

**Keywords:** teacher, stress, cortisol, alpha-amylase, ratio, ambulatory assessment

## Abstract

**Introduction:**

This longitudinal study investigates to what extent salivary alpha-amylase (as an index of the activation of the sympathetic nervous system, SNS), salivary cortisol (as an index of the activation of the hypothalamic-pituitary-adrenal, HPA, axis), and their ratio (reflecting asymmetry between both physiological stress systems) are valid indicators of stress in teachers. Moreover, we pose the question of whether dysregulation of the SNS and HPA axis is associated with individual risk and protective factors of teachers.

**Methods:**

Self-report questionnaires were used to assess personality factors, coping strategies, and perceived psychological and psychosomatic strain, with the latter being reassessed two years later.

**Results:**

The results show that cross-sectionally, alpha-amylase is positively associated with individual risk factors and psychological strain in teachers, whereas cortisol showed no significant correlations. Longitudinally, however, the ratio of alpha-amylase over cortisol was the most consistent indicator of stress in teachers, with higher values predicting a more unfavorable stress experience and psychosomatic strain.

**Discussion:**

In summary, an asymmetry between activity of the SNS and the HPA axis validly indicates work stress and psychosomatic strain in teachers.

## 1 Introduction

Many studies report that teachers feel overly stressed compared to other professions (1, 2). Teaching is demanding as it involves possibly challenging social situations characterized by unpredictability, such as dealing with unmotivated students, classroom disruptions, or providing learning opportunities for students with different skills. Psychologically, acute stress occurs when perceived situational demands (i.e., stressors) exceed perceived resources for coping and are thus perceived as threatening (3), leading to psychological and physiological responses. Physiologically, stress disturbs an organism’s homeostasis (i.e., the physiological processes that maintain an organism’s stable state; (4)). The physiological stress response enables the organism to cope with a stressor by increasing the body’s arousal and supplying energy. It includes primarily two systems: the sympathetic branch of the autonomous nervous system (SNS) and the hypothalamic-pituitary-adrenal (HPA) axis. Once stress is over, the response is deactivated, and homeostasis is restored (5). In the case of a short-term acute stressor, this adaptive reaction does not pose a problem for the organism. However, stress in everyday life is not always linear, with a clear beginning, end, and subsequent recovery. Rather, stressors often have no clear ending and occur together (6).

Chronic exposure to stressors requires ongoing adaptation, and chronic stress can change the neural brain architecture responsible for the stress reaction in a way that may lead to more maladaptive reaction patterns in response to future acute stressors (6). In the long run, this process can cause the aforementioned stress systems to fall out of balance. Empirically, dysregulations of the physiological stress response have been shown to be associated with disease states (7). Moreover, prolonged exposure to stressors could also increase a person’s psychological and psychosomatic strain. The association of this dysregulation with disease and psychological/psychosomatic strain might depend on individual characteristics such as personality factors and coping strategies that can either protect from or foster long-term stress consequences. However, the interplay between the SNS/HPA axis and their associations with individual characteristics as well as psychological strain are not fully understood.

### 1.1 The physiological stress reaction

Immediately after perceiving a stressor threatening an organism’s homeostasis, the SNS is activated. Activation of the SNS leads to the release of both adrenaline and noradrenaline (7). Adrenaline and noradrenaline release provides the body with additional energy to increase alertness and attention helping to prepare for the so-called fight-or-flight reaction (8). In addition, the activity of the parasympathetic branch of the autonomous system (i.e., the rest-and-digest reaction) is decreased. Characteristically, activation of the SNS results in an elevated heart rate and blood pressure, accelerated respiration, and constricted digestion. Activation of the SNS occurs rapidly and returns to normal as soon as the source of stress is resolved (9).

In contrast, activation of the HPA axis is slower. It involves the release of a cascade of hormones, resulting in a release of cortisol into the bloodstream. Unbound cortisol (~5-10%) has various effects on its target systems throughout the body, aiming to supply additional energy to the organism (4). Notably, peak cortisol secretion is observable between fifteen and thirty minutes after the onset of the stress reaction and about fourteen minutes after the peak activation of the SNS (10). Moreover, the HPA axis can be active in anticipation of a stressor as well as chronically (11).

Both the SNS and the HPA stress reactions can be measured by saliva samples, namely salivary alpha-amylase (sAA; 12, 13) and salivary cortisol (sC; 14). Alpha-amylase is a salivary enzyme that hydrolyses starch to glucose and maltose in the oral cavity. In recent years, sAA has been investigated as a non-invasive biomarker to assess activation of the autonomic nervous system, more specifically, of the SNS. Research has shown alpha-amylase to be highly sensitive to stress-related changes (13).

### 1.2 The interplay of the SNS and the HPA axis

For the organism to adaptively cope with acute stress, it can be assumed that the SNS and the HPA axis interact and coordinate. Anatomically, both systems intersect at the level of the central nervous system (9). However, the details of their interplay are not yet fully understood.

Due to its complexity, the interaction between SNS and HPA axis is most often studied in acute stress conditions using laboratory stress tasks such as the Trier Social Stress Test (TSST; 15). Intuitively, a coherent response of both systems would seem optimal, such that the perception of a stressor activates both the SNS and the HPA axis. Accordingly, research has shown that levels of sAA and sC throughout a stressful situation in the laboratory were reliably correlated at different time lags (10). However, studies using pharmacological suppression of either system also show an active mechanism of mutual compensation. Partial suppression of the HPA-axis stress response in an acute stress paradigm increased SNS activity compared to a placebo group (16). Conversely, suppressing SNS activity was associated with increased sC levels after stress exposure (16). Thus, the active system seems to compensate for the suppressed system (9).

An asymmetry between the SNS and HPA axis indicating dysregulation can be observed without any pharmacological suppression, i.e., with chronic stress experiences. Studies assessing stress reactivity have shown that a stressful childhood may be associated with an asymmetry in the reaction of SNS and the HPA axis. An exposure to traumatic stressors in childhood can also affect the brain significantly (6, 17). Gordis et al. (18) found an asymmetric relationship between sAA and sC in maltreated youths compared to a control group, such that SNS and HPA axis measures in reaction to the TSST were positively correlated in a control group but not among maltreated youth.

To further examine the interplay between the SNS and the HPA axis, Ali and Pruessner (19) calculated the ratio of sAA over sC as an index of asymmetry. This ratio serves as a measure that includes markers for both SNS and HPA axis reactivity as well as their balance. The authors found that adults with a history of early life adversity (ELA) showed greater asymmetry (i.e., a higher ratio of sAA/sC) in reaction to the TSST compared to adults without ELA. Moreover, the sAA/sC ratio was more strongly related to ELA as well as chronic stress and depressive symptoms compared to either sAA or sC alone. Thus, stressful events in childhood seem to be associated with a dysregulated physiological stress response, and this asymmetry can be captured in a ratio. However, studies assessing SNS and HPA axis activity in natural settings instead of stress reactivity in laboratory settings are rare. Karhula et al. (20) studied differences in sC and sAA levels in healthcare professionals with either high or low job strain with a laboratory stress task (i.e., the TSST) as well as in the field (i.e., shift work). They found that individuals with high levels of job-strain had a higher sC reactivity in the TSST compared to the group with low job-strain. Moreover, subjects working in the morning shift also had higher sAA levels 30 minutes after awakening compared to the low job strain group. However, the two groups did not differ in the sAA/sC ratio or the sC/sAA ratio. Nislin et al. (21) investigated early childhood professionals in Finland. They assessed five saliva samples per working day and one weekend day, as well as burnout symptoms and self-reported work engagement. Exploring the sAA/sC ratio and the sC/sAA ratio, they did not find significant correlations between either ratio values and self-reported work engagement nor between ratio values and burnout. However, they noted that their specific sample scored lower on burnout compared to teachers in general education and other professions. Thus, the relationship between the physiological stress reaction and ongoing work stress is less clear.

### 1.3 Physiological stress reactions and stress consequences

Individuals vastly differ in stress reactivity levels, and stress reactivity is often speculated to be related to susceptibility to different diseases. Individual susceptibility to cardiovascular disease is linked to cardiovascular stress responses, such that greater stress reactivity and poorer recovery from acute mental stressors predict future cardiovascular disease (22). A recent review by Turner et al. (23) showed that this reactivity hypothesis also seems true regarding other biomarkers (e.g., blunted salivary cortisol reactivity), and stress consequences (e.g., regulatory T cell percentage). Moreover, they assume an invented-U model, in which both an exaggerated and a blunted stress response are related to adverse longitudinal physiological health consequences. However, in this overview, only two studies included assessed markers of both physiological systems and longitudinal physiological health outcomes, with inconclusive results.

Psychological stress consequences and their association with the physiological stress response are less clear. A coherent response of all involved stress systems (i.e., the psychological system or either of the two physiological stress systems) is often considered adaptive, meaning that a stressor concurrently activates both, the psychological processing of the stressful stimulus and the physiological stress response. Accordingly, once the stress is averted, all systems deactivate and recover. However, research shows that activation of the psychological and the physiological system is not strongly associated (24, 25). Even if the physiological stress reaction is pharmacologically suppressed, the emotional stress reaction still occurs (26). Thus, the psychological and physiological stress systems appear to function almost independently of each other. Accordingly, people often do not consciously perceive their own physiological stress reactions. If the physiological arousal is perceived, it is often misattributed to a different source and not to the acute stressor. Experiencing psychological stress therefore appears to depend on other factors and not necessarily on physiological arousal. However, if the short-term psychological stress reaction is practically independent of the physiological arousal, one could speculate that any longer-term psychological stress consequences (e.g. psychological strain or psychosomatic consequences) are also not necessarily related to the physiological dysregulation. However, this association is unclear.

Psychological strain can manifest in different areas of life (e.g., at work, regarding the self, or in one’s private life; 27). Occupational problems also include negative feelings related to work. Self-related problems include low frustration tolerance, feelings of depersonalization, or concentration problems. Family-related problems encompass estrangement or decreased participation in familial life. Friends-related problems include reduced interest in friends’ lives and retreating from social contact. Psychological strain can also include psychosomatic symptoms. Vital exhaustion is a psychosomatic state of unusual fatigue, lack of energy, irritability, and demoralization (28). Vital exhaustion is a potential early warning sign of cardiovascular disease (29) and seems closely related to burnout (30).

### 1.4 Individual characteristics and the stress response

Individual personality differences can influence stress reactivity (31) and might influence whether stress consequences are evident psychologically. A high core self-evaluation (CSE) can protect an individual against stress (32, 33). CSE is a higher-order construct that includes four personality traits: global self-esteem, generalized self-efficacy, emotional stability, and locus of control. People with high CSE perceive themselves as worthy, capable, and in control of their lives (34). In contrast, neuroticism (i.e., emotional instability) is the disposition to experience negative affectivity (e.g., anger, anxiety, self-consciousness, irritability, depression; (35), and people with high neuroticism interpret ordinary situations as more threatening (36).

Individuals also differ in their strategies to cope with a perceived stressor. However, not all strategies are adaptive in the long run. Approach strategies involve actively confronting the stressor (e.g., active problem-solving, seeking support). In contrast, avoidance strategies aim to withdraw from the stressor (e.g., giving up, social withdrawal), but the problem remains unsolved. Thus, while successful in the short term, the unresolved situation might continue to generate stress. Both personality traits and coping strategies can influence how a stressor is dealt with.

### 1.5 The present study

Teaching involves many challenging situations to which the teacher ideally reacts proactively. A high density of social interactions with students, colleagues, or parents, social exposure and evaluation, and classroom disruptions can all contribute to teacher stress (37). However, to the best of our knowledge, no studies have assessed the association between teacher stress and the dysregulation of the physiological stress reaction, so far.

The dysregulation of the physiological stress response has mainly been investigated in laboratory settings using (acute) stress tasks such as the TSST, but not in response to frequently occurring and/or chronic stressors so that results cannot be easily generalized to everyday life. Social settings differ substantially with respect to stress reactions. Thus, particularly for a socially demanding profession such as teaching, physiological stress-sensitive responses should be measured using ambulatory assessments (i.e., during teaching and during leisure time).

Moreover, most studies rely on one or two stress-related outcomes. Even if different concepts of psychological and psychosomatic strain are related, they differ in their underlying models and operationalizations. Thus, it is important to include heterogeneous constructs to assess the consequences of psychological stress in different areas of life. Additionally, individual characteristics such as personality factors and coping styles can serve as risk or protective factors.

To this end, we measured teachers’ physiological stress-sensitive markers, namely sAA and sC, on two workdays and one leisure day. To incorporate both stress systems, we used the sAA/sC ratio to explore a possible dysregulation of the SNS and the HPA axis and associations with personality factors, coping styles, and psychological or psychosomatic strain. Moreover, we assessed teachers’ psychological and psychosomatic strain on two time points, that is, at baseline, and at 2-year follow-up, to consider longitudinal aspects. We pose the following research questions:

1. How are the two stress systems SNS and HPA-axis and their dysregulation associated with teachers’ personality factors, coping styles, and psychological strain?
2. Do the associations differ depending on the setting (workdays vs. one leisure day)?
3. How are the two stress systems SNS and HPA-axis and their dysregulation related to the psychological strain measured two years later?

## 2 Method

This study is part of a larger project examining psychobiological stress in teachers (38, 39) and includes a secondary analysis of previously published data of sAA and sC (40).

### 2.1 Sample

The study included 42 apparently healthy teachers (28 female) at baseline. The average age was 39.66 years (SD = 11.99). Participants were recruited via flyers and circular emails sent to school administrations in the canton of Bern, Switzerland. After the first measurement, one teacher moved abroad, and two teachers withdrew their participation due to pregnancy, resulting in a sample of 39 teachers two years later. All teachers were screened for the inclusion and exclusion criteria during a short telephone interview. Inclusion criteria for participation in the study were employment as a primary or secondary teacher in the Swiss canton of Bern and a workload of at least 16 lessons per week (equivalent to at least 60 percent of full-time employment). Exclusion criteria were working outside of the canton of Bern, acute infections, cardiovascular or other chronic diseases, use of cardiovascular drugs or other medication in the past two months (except phytopharmaceuticals), substance abuse, drug use in the last four weeks, more than two standard units of alcohol per day, smoking more than ten cigarettes per day, long-distance flights within the last two weeks, and pregnancy. Participants worked at 39 schools and reported a mean of 13.35 years of teaching experience (SD = 11.07, range = 1–40). Participants had completed standard teacher training and were regular classroom teachers, not teachers for the gifted or students with special needs. The grade levels teachers taught ranged from kindergarten and elementary school (kindergarten to 6th grade; n = 27), over secondary school (7th to 9th grade; n = 12), to high school and vocational school (10th to 12th grade; n = 3). Enrolled participants provided informed consent. The study was approved by the ethics committee of the canton Bern (no. 2019–00787), as well as the Internal Review Board (IRB) of the University of Bern. It was conducted in strict compliance with current data protection laws.

### 2.2 Design and procedure

The study comprised a longitudinal multi-method ambulatory design. Assessments were conducted at three time points: Participants completed online questionnaires on personality factors, coping styles, and psychological strain at t0 in December 2019 (before the Covid-19 pandemic).

Saliva samples were collected on two workdays and one leisure day in an ambulatory assessment design between January and November 2020 (t1; before and during the pandemic). When saliva samples were collected, teachers taught in person, not online. The teachers were instructed verbally and in writing to refrain from drinking alcohol starting the evening before each collection day. They were further instructed not to engage in strenuous physical activity on the three ambulatory assessment days. The three saliva collection days for each participant took place within one week. The order (workday or leisure day first) was randomized. However, due to rescheduling among two participants, 23 teachers first collected the leisure day samples, while 19 started with the workday sample collection. When determining the data collection days, we considered the menstrual cycle of our female participants to ensure that the three days of data collection did not fall within the follicular phase, as this could affect cortisol binding and HPA axis reactivity (14).

Two years later (t2), the psychological strain was reassessed using online questionnaires.

### 2.3 Measures

#### 2.3.1 Self-reports

Participants completed questionnaires assessing individual characteristics (personality factors, coping styles), their levels of perceived psychological and psychosomatic strain, and their general stress level.

To assess personality, participants completed questionnaires on CSE and neuroticism. CSE was assessed with the German version (41) of the original Core Self-Evaluation Scale (42). Cronbach’s alpha of this sample was α = .81. Neuroticism was measured by two items of the German short version of the BFI-10 (43) and two items of the German NEO-FFI (44). Cronbach’s alpha of the four items was α = .73 (see Schneider et al. (40) for details of scale construction).

Coping strategies were assessed with the German Measure of Coping Capacity Questionnaire (MECCA; 45). The MECCA comprises eleven behavior and experience patterns, three of which can be described as coping strategies in a narrow sense (i.e., the ability to distance oneself (α = .86), proactive problem solving (α = .80), resignation tendency (α = .84)). Further, we constructed two items to assess the tendency to seek out positive experiences (“I take time to do things that are good for me”; “I often don’t have time for my favorite activities [reversed]”). Participants responded on a scale from 1 (does not apply at all) to 5 (does apply entirely).

To assess psychological and psychosomatic strain, participants completed the German Burnout Screening Scales (BOSS) I and II, consisting of subscales assessing occupational problems (t0: α = .91; t2: α = .83), self-related problems (t0: α = .91; t2: α = .91), family-related problems (t0: α = .81; t2: α = .83), friends-related problems (α = .70; only assessed at t0), and physical problems (α = .74; only assessed at t0) (27). Further psychosomatic symptoms were evaluated using the Maastricht Vital Exhaustion Questionnaire (MQ; 46) in its German translation (47). Cronbach’s alpha for this sample was α = .88 at t0 and α = .91 at t2.

In addition, two items were constructed to assess general work stress (“Please rate the degree of your work stress”; 1 (very low) to 5 (very high)) and health problems (“How would you rate your state of health recently?”; 1 (very well) to 5 (very bad)).

#### 2.3.2 sAA and sC

To assess sAA and sC, saliva was repeatedly collected on two workdays and one leisure day. On the workdays, participants were asked to collect saliva at the following time points: wake-up, + 30 min, +45 min, 8 am, 10 am, 12 am, 4 pm, and 8 pm. On the leisure days, aiming at equivalent measurement time points, participants were asked to collect saliva samples at the following time points: wake-up, + 30min, +45min, +2h, +4h, +6h, +10h, and +14h. Participants were asked to enter the time of sampling in a log booklet. On the workdays, teachers woke up between 3.45 a.m. and 7.00 a.m. On the leisure day, wake-up time ranged from 4.37 a.m. to 9.55 a.m.

Participants were instructed verbally and in writing to gently chew cotton rolls (Sarstedt, Sevelen, Switzerland) for 2 minutes before placing them back in the provided container. After collection, participants stored the samples in their home freezer until a member of the research team picked them up and kept them in a study freezer at -20˚C at the University of Bern until biochemical analyses took place at the biochemical laboratory of the Psychological Department of the University of Zurich, Switzerland. Samples were thawed and centrifuged at 3000 rpm for 10 minutes. Afterward, free sC (nmol/L) was analyzed using an immunoassay with time-resolved fluorescence detection (48), while the activity of sAA (Unit/mL) was analyzed using a kinetic colorimetric test (49).

### 2.4 Statistical analysis

All data were analyzed using IBM SPSS Statistics (IBM SPSS Statistics, Version 28). For the self-reported measures, normal distribution was tested using the Shapiro-Wilk test. Skewed variables were then log-transformed using the natural logarithm. Potential confounders, such as age, sex, and teaching experience, were tested for significant associations with the main variables by calculating Pearson correlations. No significant associations were found.

To measure the overall intensity of sAA and sC, the area under the curve with respect to ground (AUCg) was calculated (50) using the raw, untransformed values of the five measurement points from 8 am to 8 pm (resp. +2h and +14h of waking up at the leisure day). AUCg represents both the baseline and the diurnal course in one score. For both sAA and sC, AUCg was calculated twice: first, using the averaged measures of the two workdays, and second, for the leisure day. As the calculated AUCg values were not normally distributed, they were log-transformed using the natural logarithm.

To assess the dysregulation of the SNS and HPA axis, the ratio of sAA over sC was calculated using the calculated AUCg (not yet log-transformed; see Sollberger and Ehlert (51) for a discussion of how to calculate a ratio). The ratio was calculated twice: first, using the AUCg of both sAA and sC of the averaged workdays, and second for the leisure day. Then, according to the procedure Sollberger and Ehlert (51) described, we log-transformed both ratios using the natural logarithm.

We calculated descriptive statistics of all variables and bivariate Pearson correlations. To account for the different saliva collection times, we controlled for the time delay between psychometric assessment at t0 and saliva collection. To assess the longitudinal associations between physiological measures and psychological strain at t2, we calculated partial correlations controlling for the psychological strain variables of t0. A Wilcoxon signed-rank test was performed to investigate any significant differences in the ratio of the averaged workdays and the leisure day. All analyses were two-tailed, with the significance level set at p <.05.

## 3 Results

### 3.1 Descriptive statistics

Descriptive statistics for the physiological stress-sensitive measures (AUCg of sAA and sC, and their ratio) are depicted in **Table 1**. Results regarding the diurnal course and the awakening reaction of both alpha-amylase and cortisol are reported elsewhere (40). Intercorrelations are reported in the **Appendix (Table A1)** and in detail in Wettstein et al. (39).

**Table 1:** Descriptive statistics of the physiological measures.

Measures (AUCg)	n	Mean	SD	Min	Max
sAA (Unit/mL x time)					
workday 1	41	1480.66	1184.59	45.35	4435.86
workday 2	42	1309.41	1227.41	31.78	5401.19
averaged workdays	41	1390.36	1015.26	179.16	4095.45
leisure day	37	1459.53	1007.17	139.80	3948.82
sC (nmol/L x time)					
workday 1	41	34.19	16.68	11.84	83.66
workday 2	42	45.86	54.32	13.58	377.62
averaged workdays	41	39.94	28.51	12.71	200.30
leisure day	37	30.88	10.81	11.89	64.41
sAA/sC ratio					
averaged workdays	41	45.29	43.59	4.45	167.34
leisure day	37	57.20	52.52	4.71	258.96
sAA/sC ratio logarithmized					
averaged workdays	41	3.38	0.99	1.49	5.12
leisure day	37	3.64	0.98	1.55	5.56

Depicted are the descriptive statistics of the raw area under the curve with respect to the ground (AUCg) of salivary alpha-amylase (sAA) and salivary cortisol (sC) measures, as well as the descriptive statistics of the calculated ratio sAA/sC, before and after log-transformation. The ratio averaged workdays was calculated as sAA (averaged workdays) over sC (averaged workdays). Similarly, the ratio leisure day was calculated as sAA (leisure day) over sC (leisure day).

### 3.2 The difference in the ratio between averaged workdays and leisure days

A Wilcoxon signed-rank test was performed to investigate differences in the assessment setting of the physiological measures sAA, sC, and their ratio. Results revealed no significant difference between sAA of the averaged workdays (Mdn = 1029.17) and sAA of the leisure day (Mdn = 1443.16, z = -0.57, p = .572), as well as no significant difference between the sAA/sC ratio of the averaged workdays (Mdn = 3.34) and the sAA/sC ratio of the leisure day (Mdn = 3.74, z = -1.40, p = .162). However, there was a significant difference between sC of the averaged workdays (Mdn = 35.91) and sC of the leisure day (Mdn = 29.85, z = -1.98, p = .048, r = .33).

### 3.3 Bivariate correlations

Bivariate correlations between assessed psychological variables and the physiological markers on workdays are depicted in detail in **Table 2**.

**Table 2:** Bivariate correlation coefficients of physiological markers assessed at the workdays (averaged) and psychometric variables assessed at t0 and t2.

Variable	Physiological measures (workdays averaged)		
	sAA AUCg	sC AUCg	sAA/sC
Sex	.02	.28	-.12
Age	-.02	.01	-.02
Core Self-Evaluation	-.43**	-.01	-.36*
Neuroticism	.41**	-.11	.40*
Seeking out positive experiences	-.40*	.07	-.37*
Resignation tendency	.30	.27	.14
Ability to distance oneself	-.14	-.08	-.08
Proactive problem solving	-.11	-.02	-.08
Occupational problems t0	.24	.17	.12
Self-related problems t0	.45*	.05	.36*
Family-related problems t0	.25	.07	.18*
Friends-related problems	.40*	-.01	.34*
Physical problems	.37*	-.07	.35*
Vital exhaustion t0	.36*	-.03	.32*
Work stress t0	.01	.26	-.11
Health problems t0	.26	-.05	.25
Occupational problems t2	.35*	-.43	.49**
Self-related problems t2	.22	-.25	.30
Family-related problems t2	.04	.02	.02
Vital exhaustion t2	.22	-.42*	.38*
Work stress t2	.50**	-.25	.53***
Health problems t2	.30	-.30	.39*

AUCg, area under the curve with respect to the ground; sAA, salivary alpha-amylase; sC, salivary cortisol. Variables at t0 were controlled for the time delay between psychometric assessment of t0 and saliva sampling (t1). Variables at t2 were controlled for delay, and variables at t0. * p <.05. ** p <.01. ***p <.001.

#### 3.3.1 Individual characteristics and psychological or psychosomatic strain at t0

On the averaged workdays, sAA was moderately but almost consistently associated with personality factors, psychological and psychosomatic strain measures at t0. Results showed negative correlations with two protective factors (CSE, seeking out positive experiences), while correlations with neuroticism and psychological and psychosomatic strain variables were positive. However, there were no significant associations between sAA and the coping styles resignation tendency, proactive problem solving and the ability to distance oneself. Results also showed no significant correlations between sC and any measures at t0. The sAA/sC ratio was consistently associated with risk and protective factors as well as with psychological and psychosomatic strain measures, mirroring the correlations of sAA. To further explore whether the significant correlations found with sAA and the sAA/sC ratio differ in strength, Williams’ tests for dependent correlations were conducted. However, there were no statistically significant differences between the correlations found.

In contrast, there were no significant correlations between the physiological markers assessed on the leisure day and any measure assessed at t0 (all p >.05), except for vital exhaustion, which positively correlated with sC (r(39) = .34, p = .042). See **Table A1** in the Appendix for details on bivariate correlations of physiological measures assessed on the leisure day.

#### 3.3.2 Psychological and psychosomatic strain at t2

sAA of the averaged workdays was solely and positively associated with both occupational problems and work stress at t2, while sC of the averaged workdays was significantly negatively related to occupational problems and vital exhaustion at t2. More consistently, the sAA/sC ratio was significantly positively associated with four out of six variables (i.e., occupational problems, work stress, vital exhaustion, and health problems) at t2. To further explore any differences in strength, Williams’ tests for dependent correlations were conducted. There were no significant differences in strength between the found correlations, with two exceptions: The correlations between occupational problems at t2 and sAA and the sAA/sC ratio differed significantly (t(38) = -2.16, p = .037). Moreover, the correlation between vital exhaustion at t2 and sC and vital exhaustion at t2 and the sAA/sC ratio differed significantly, t(38) = -3.23, p = .003.

In contrast, there were no significant associations between any physiological marker assessed on the leisure day and psychological and psychosomatic strain variables at t2, except for a significant association between sC and work stress (r(35) = .36, p = .044).

## 4 Discussion

This study examined the interplay of two components of the physiological stress response in teachers, the SNS and HPA axis activation, and its associations. Several characteristics of the teaching profession may contribute to the finding that teachers are more stressed than other professionals. Among others, teaching involves many situations that are not predictable (e.g., student misbehavior or aggression) which might be perceived as threatening by the teacher. We aimed to understand how markers of teachers’ physiological stress systems, that is, salivary alpha-amylase (sAA), salivary cortisol (sC), and their dysregulation (sAA/sC ratio), are associated with personality factors, coping strategies, as well as psychological and psychosomatic strain. The overall intensity of alpha-amylase and cortisol secretion was operationalized with the area under the curve with respect to the ground (AUCg), which represents the baseline and diurnal course in one score. To investigate differences due to the setting, we assessed teachers’ stress-sensitive physiological markers on two workdays and on a leisure day. Moreover, two years later, we reassessed psychological and psychosomatic strain to study longitudinal associations between the above.

Results show that on the averaged workdays, especially sAA is cross-sectionally associated with individual characteristics and both psychological and psychosomatic strain measures. The sAA/sC ratio mirrors these associations. Differently, sC is associated with neither individual characteristics nor with psychological and psychosomatic strain at t0. In contrast to the findings on workdays, physiological markers assessed on the leisure day do not show any significant association with measures at t0, except for a positive association between vital exhaustion and sC. Thus, under non-working conditions, there does not seem to be a consistent link between stress-sensitive biomarkers and psychological and psychosomatic strain measures or with individual characteristics. This suggests that factors specific to the teaching profession were at least partly responsible for our results on averaged workdays. However, we did not find a significant difference between averaged workdays and the leisure day regarding sAA or the sAA/sC ratio. However, the sC AUCg of the averaged workdays significantly differed from the AUCg of the leisure day. On workdays, teachers seemed to have moderately increased levels of sC compared to a leisure day.

Thus, elevated diurnal sAA levels could indicate increased levels of perceived psychological and psychosomatic strain. However, as the data reported in this study is correlational, the direction of the association is unclear. Elevated sAA on workdays could also lead to increased psychological or psychosomatic strain. Results also show significant associations of personality with sAA as well as with the sAA/sC ratio. Thus, elevated diurnal sAA levels were associated with low CSE and high neuroticism. This adds to the few existing studies that demonstrate a positive correlation of neuroticism and basal sAA (52) or its reactivity to psychological stress (53). However, associations with coping strategies are less clear in our results. Only the seeking of positive experiences was negatively related to sAA (but not the sAA/sC ratio or sC). This might be because coping strategies rely on the individual’s perception of a situation as stressing (e.g., on the psychological level). However, physiological and psychological stress processing is often dissociated (24). More research is needed to better understand coping behavior and physiological stress responses.

Longitudinally, results also show significant correlations between the averaged stress-sensitive markers assessed on workdays and psychological and psychosomatic strain assessed two years later. sAA was positively correlated with occupational problems and work stress, whereas sC showed negative correlations with occupational problems and vital exhaustion. Moreover, the sAA/sC ratio was related to the psychological and psychosomatic strain measures at t2 positively and almost consistently. In contrast, there were no associations between any measure at t2 and physiological markers assessed on the leisure day (with one exception). Again, this suggests that the found associations between psychological and psychosomatic strain at t2 and physiological markers assessed on the workdays are linked to work stress-related reactivity of the physiological stress systems rather than other possibly occurring stressors.

These results could indicate that in relation to long term stress consequences, the ratio might serve as a better marker than either sAA or sC alone. Though most differences between dependent correlations were not statistically significant, this was not as surprising considering the small sample size. However, the consistency in the results seems promising and requires further exploration. The ratio could be interpreted as indicator of asymmetry between the two physiological stress systems. This suggests that an asymmetry between the SNS and HPA axis in reaction to stressors at work could lead to more perceived long-term psychological strain. Contrary to our findings, Nislin et al. (21) found no significant correlations between ratios and self-reported work engagement or burnout in early childhood professionals. However, their specific sample scored lower on burnout symptoms compared to teachers in general education, and they noted that links between physiological stress and psychological work-related stress variables might require more extreme emotional states to be reflected in changes in stress hormones.

We did not find significant associations of the AUCg of sC and any measure at t0, and only negative correlations of sC and psychological and psychosomatic strain at t2. In fact, research linking HPA axis functioning to chronic stress variables is inconclusive. Rather, both elevated and blunted HPA responses have been reported, depending on the studied construct and the methodology (54). Thus, it is possible that blunted cortisol in response to work stress is associated with higher levels of psychological and psychosomatic strain at t2. This is also in line with other results obtained with the same sample of teachers. La Marca et al. (38) found no statistically significant associations between hair cortisol and self-reported psychological strain, with only one exception. In contrast, more objectively assessable stressors (e.g., observed student aggressions) did show positive associations with hair cortisol (38). Due to this lack of significant associations between cortisol and psychological strain in our results, we examined in additional analyses (not reported here) whether salivary cortisol’s daily slope and the awakening response as indicators of high or blunted cortisol release throughout the day (40) were associated with psychological strain. However, we found no statistically significant associations. Thus, more research is needed to better understand this relationship.

Taken together, these results indicate that teachers’ physiological SNS and HPA activity on workdays, but not on a leisure day, are related to perceived psychological and psychosomatic strain, both cross-sectionally and longitudinally. Especially longitudinally, both physiological stress systems and their interplay need to be investigated further, as a possible dysregulation can have adverse effects, which can also manifest on a psychological level by increasing problems across a wide range of life areas.

### 4.1 Limitations and strengths

The present study has several limitations. First, the sample was relatively small and consisted of apparently healthy and medication-free teachers, limiting generalizability. Second, the saliva sampling relied solely on our participant’s accuracy in following the sampling instructions, as there was no objective control of compliance. Thus, it is possible that the results were influenced by non-adherence to the sampling protocol, limiting their ecological validity. Third, participants gave stimulated (chewing on cotton rolls) in contrast to non-stimulated (passive drooling) saliva samples. This could have possibly altered results concerning sAA, as different salivary glands may have different salivary secretion rates, which could influence the amounts of sAA delivered into the oral fluid (55, 56). Fourth, there is some evidence that sAA can also be influenced by parasympathetic activity (56). Thus, it might not be a pure marker of SNS activity. Fifth, salivary measures were assessed before and during the COVID-19 pandemic. Although teachers were teaching face-to-face in the classroom as usual during the data collection period, we cannot exclude that factors related to the pandemic might have influenced the results. While it is still interesting that most of the associations were only statistically significant using workday saliva samples, we did not control what kind of stressors led to this result. Thus, the reported results should be interpreted accordingly. Last, our results are based on correlations and should not be interpreted causally. Future research should replicate the presented finding with a larger, less homogenous sample.

Nevertheless, this study has several strengths. First, the combination of self-reports and an ambulatory assessment of stress-sensitive biomarkers increases the ecological validity of the presented results. Few studies assess the response of the physiological stress systems outside of the laboratory. However, ambulatory assessment is necessary to understand stress occurring in natural settings (57). Moreover, the longitudinal assessment of psychological strain allows for a more nuanced statement regarding long-term stress consequences. Thus, this study contributes to the scarce literature on physiological stress in natural settings and its (longitudinal) associations with strain. Second, whereas much is known about cortisol, alpha-amylase and its associations are less studied. Moreover, the ratio of alpha-amylase and cortisol promises to be a lean way to assess a possible asymmetry of the physiological stress systems. Thus, the present study can help researchers to identify important factors linked to alpha-amylase and the SNS.

### 4.2 Conclusion

The present study combines a longitudinal multi-method design with ambulatory assessment and provides crucial information on the interplay of the two physiological stress systems, the SNS and HPA axis, in teachers. Results show that, cross-sectionally, sAA was an important indicator of increased levels of psychological and psychosomatic strain and was positively related to individual characteristics of risk. Longitudinally, the sAA/sC ratio as a reflection of an asymmetry between sAA and sC was positively related to psychological and psychosomatic strain. These results underline the importance of including measurements of both physiological stress systems. Moreover, the physiological response becomes evident also on a psychological level in that it is related to higher levels of strain.

## Data Availability

The raw data supporting the conclusions of this article will be made available by the authors, without undue reservation.

## Appendix

Table A1. Intercorrelations of saliva measures and the ratios.

**Table:**

Variable	1	2	3	4	5	6	7	8	9	10	11	12
1. Sex	–											
2. Age	.35*	–										
3. AUCg sAA WD1	.09	-.05	–									
4. AUCg sAA WD2	-.02	.08	.39*	–								
5. AUCg sAA WD-A	-.01	-.04	.79**	.81**	–							
6. AUCg sAA LD	.01	.01	.14	.41*	.33	–						
7. AUCg sC WD1	.04	.05	-.20	.22	-.03	.08	–					
8. AUCg sC WD2	.38*	.02	.08	-.21	-.11	.11	.15	–				
9. AUCg sC WD-A	.29	.02	-.04	-.06	-.10	.14	.57**	.88**	–			
10. AUCg sC LD	-.15	-.15	.46**	.16	.32	-.24	-.19	-.02	-.10	–		
11. sAA/sC ratio WD-A	-.15	-.04	.69**	.72**	.89**	.22	-.28	-.49**	-.54**	.32	–	
12. sAA/sC ratio LD	.06	.07	-.06	.28	.15	.93**	.14	.10	.15	-.58**	.06	–

AUCg, area under the curve with respect to the ground; sAA, salivary alpha-amylase; sC, salivary cortisol; WD1, workday 1; WD2, workday 2; WD-A, workdays averaged; LD, leisure day. **p* <.05. ***p* <.01.

**Table A2.** Bivariate correlation coefficients of physiological markers assessed at the leisure day and variables assessed at t0 and t2.

**Table:**

Variable	Physiological measures (leisure day)		
	sAA AUCg	sC AUCg	sAA/sC
Sex	.02	-.16	.07
Age	.02	-.16	.08
Core Self-Evaluation	-.30	-.18	-.18
Neuroticism	.25	.20	.13
Seeking out positive experiences	-.18	-.16	-.09
Resignation tendency	.14	.33	-.01
Ability to distance oneself	.05	-.09	.07
Proactive problem solving	-.03	-.21	.05
Occupational problems t0	.03	.27	-.08
Self-related problems t0	.12	.28	.00
Family-related problems t0	.16	.00	.13
Friends-related problems	.14	.30	.01
Physical problems	.15	.17	.06
Vital exhaustion t0	.16	**.33***	.01
Work stress t0	.02	-.08	.05
Health problems t0	.18	.32	.03
Occupational problems t2	-.12	.28	-.21
Self-related problems t2	-.26	.09	-.26
Family-related problems t2	-.06	-.05	.03
Vital exhaustion t2	-.18	.15	-.21
Work stress t2	-.15	**.36***	-.27
Health problems t2	.07	.00	.06

AUCg, area under the curve with respect to the ground; sAA, salivary alpha-amylase; sC, salivary cortisol. Variables at t0 were controlled for time delay between psychometric assessment of t0 and saliva sampling (t1). Variables at t2 were controlled for delay and variables at t0. * p <.05. ** p <.01. ***p <.001.
